# Time-dependent cytokine landscapes in an ex vivo microfluidic glioblastoma platform

**DOI:** 10.1007/s00262-026-04486-w

**Published:** 2026-07-06

**Authors:** Amr Moursi, Antonia Barry, Lauric Feugere, Alex Iles, Ian Scott, Shailendra Achawal, Chittoor Rajaraman, Pedro Beltran-Alvarez, John Greenman

**Affiliations:** 1 Centre for Biomedicine, Hull York Medical School, University of Hull, Hull, UK; 2 Neurosurgery Department, The Walton Centre NHS Foundation Trust, Liverpool, UK; 3 Cancer and Genomics Sciences at the University of Birmingham, Birmingham, UK; 4 Marine Ecological and Evolutionary Physiology Laboratory, Department of Biology, Chemistry and Geography, Université du Québec à Rimouski, Rimouski, Canada; 5 Department of Chemistry, Stockholm University, Arrhenius Laboratory, Stockholm, Sweden; 6 Neuroscience Laboratories, The Walton Centre NHS Foundation Trust, Liverpool, UK; 7 Department of Neurosurgery, Hull University Teaching Hospitals NHS Trust, Hull Royal Infirmary, Hull, UK

**Keywords:** Glioblastoma, Microfluidics, Ex vivo culture, Patient-derived biopsy, Cytokine profiling, Tumour-on-a-chip

## Abstract

Glioblastoma (GBM) is the most common and aggressive astrocytic glioma of the central nervous system with a median survival of 15 months from diagnosis. Patient outcomes have improved only marginally over the past 20 years, and GBM research is limited by models that fail to preserve the native architecture and function of tumours over time. The aim of our study was to provide a physiologically and clinically relevant microfluidic environment for the study of GBM. We designed and validated a custom-built polymethyl methacrylate (PMMA) microfluidic perfusion platform, and successfully maintained ex vivo culture of intact, fresh, patient-derived GBM biopsies for up to 12 days. Cellular viability and tissue integrity were assessed by histology, apoptosis markers, and cytokine profiling, whilst cellular stress was quantified by lactate dehydrogenase (LDH) release. LDH levels initially decreased and then remained low and stable up to 12 days. Haematoxylin and eosin staining showed that tissue architecture was preserved, with mitotic figures post-perfusion. Cleaved PARP and Annexin V immunohistochemistry revealed no significant increase in apoptosis between pre- and post-perfused tissues at 8 or 12 days. Effluent cytokine analyses demonstrated sustained secretory activity with a time-dependent modulation (across 105 analytes: *p* = 0.0288). Quantitative ELISAs for seven cytokines (VEGF, MMP9, CHI3L1, IL-6, IL-8, Serpin-E1, Angiopoietin-2) corroborated cytokine analysis trends and correlated strongly with array data (*p* < 0.001), validating assay fidelity and biological signal. Collectively, biochemical, histological and secretome readouts show that intact GBM tissues remain viable, metabolically active and analysable on this platform for up to 12 days. The system provides a practical, physiologically relevant, model for longitudinal GBM studies and lays the groundwork for scalable translational testing of patient tissue.

## Introduction

Glioblastoma (GBM) is the most common and aggressive primary malignant brain tumour in adults. Despite maximal safe resection followed by radiotherapy and concomitant temozolomide (TMZ), median survival remains only 15 months, and there has been little improvement in outcomes over the past two decades [1, 2]. GBM is characterised by marked intra-tumoural heterogeneity and diffuse infiltration into the surrounding brain. It also develops within a complex microenvironment comprising vascular, stromal and immune components, which together drive treatment resistance and recurrence [3].

Conventional preclinical models only partially recapitulate this complexity. Two-dimensional (2D) glioma cell lines are inexpensive and well-suited to high-throughput drug screening, but they lack three-dimensional architecture and cell–matrix interactions. By contrast, spheroid and organoid models provide a more physiologically relevant 3D structure and can replicate some aspects of tumour heterogeneity; however, these require relatively long culture times, usually lack vasculature and immune components, and cannot definitively reproduce the individual tumour pathology [4–7].

Animal models, including xenograft and genetically engineered mouse models, zebrafish, and spontaneous canine gliomas, have been indispensable for studying GBM biology and testing new therapies. Nevertheless, important limitations remain with these models too. Species-specific anatomical, genetic, and immunological differences mean these systems do not fully mirror human disease [8, 9]. This likely contributes to the poor translation of preclinical findings into effective therapies; consequently, there is increasing focus on human GBM tissue-based models [8, 9].

Microfluidic “tumour-on-a-chip” technologies have emerged as promising alternative platforms to bridge the gap between static in vitro/ex vivo models and the dynamic, perfused in vivo tumour microenvironment. These devices enable continuous perfusion, precise control of flow and gradients, and miniaturisation of culture volumes, thereby providing a more in vivo-like environment while reducing reagent and tissue requirements [10]. Microfluidic systems have been applied to a wide range of human tissues, including ovarian [11], lung [12], rectal [13], thyroid [14], head and neck [15], and brain [16].

In glioma and GBM specifically, early microfluidic platforms enabled the culture of glioma cell lines in 2D channels to study acute drug responses [10], while later designs incorporated 3D spheroids [17]. More advanced systems have introduced vascular-like networks and brain tumour stem-like cells to model perivascular invasion and heterogeneity [18], or focused on dynamic cell migration through constricted channels to predict progression-free survival and recurrence in GBM patients [19]. A smaller number of studies have used microfluidic perfusion to maintain patient-derived primary human GBM cells [19], or intact patient-derived tumour tissue ex vivo [20, 21]. Devices of the latter type preserve native architecture and stromal components by avoiding enzymatic dissociation, offering a closer approximation to the in situ tumour microenvironment, although are limited as to the time samples can be maintained in a viable state.

In this study, we build on previous work from our group to validate a simple, polymethyl methacrylate (PMMA)-based, microfluidic perfusion platform for the ex vivo culture of intact patient-derived GBM tissue. Fresh surgical biopsies were micro-dissected and successfully maintained under continuous low-shear perfusion for up to 12 days. Viability and structural integrity were assessed using lactate dehydrogenase (LDH) release, haematoxylin and eosin (H&E) staining and immunohistochemistry for apoptotic markers. In parallel, we performed multiplex cytokine profiling followed by targeted enzyme-linked immunosorbent assay (ELISA) validation to characterise dynamic changes in the GBM secretome over time, and in response to treatment. Our aim was to establish the feasibility and constraints of this GBM tissue-on-a-chip platform as a physiologically relevant, technically accessible model for the prolonged culture of patient-derived tumour biopsies, providing a basis for future personalised therapeutic studies.

## Methods

### Device fabrication

**Fig. 1:**
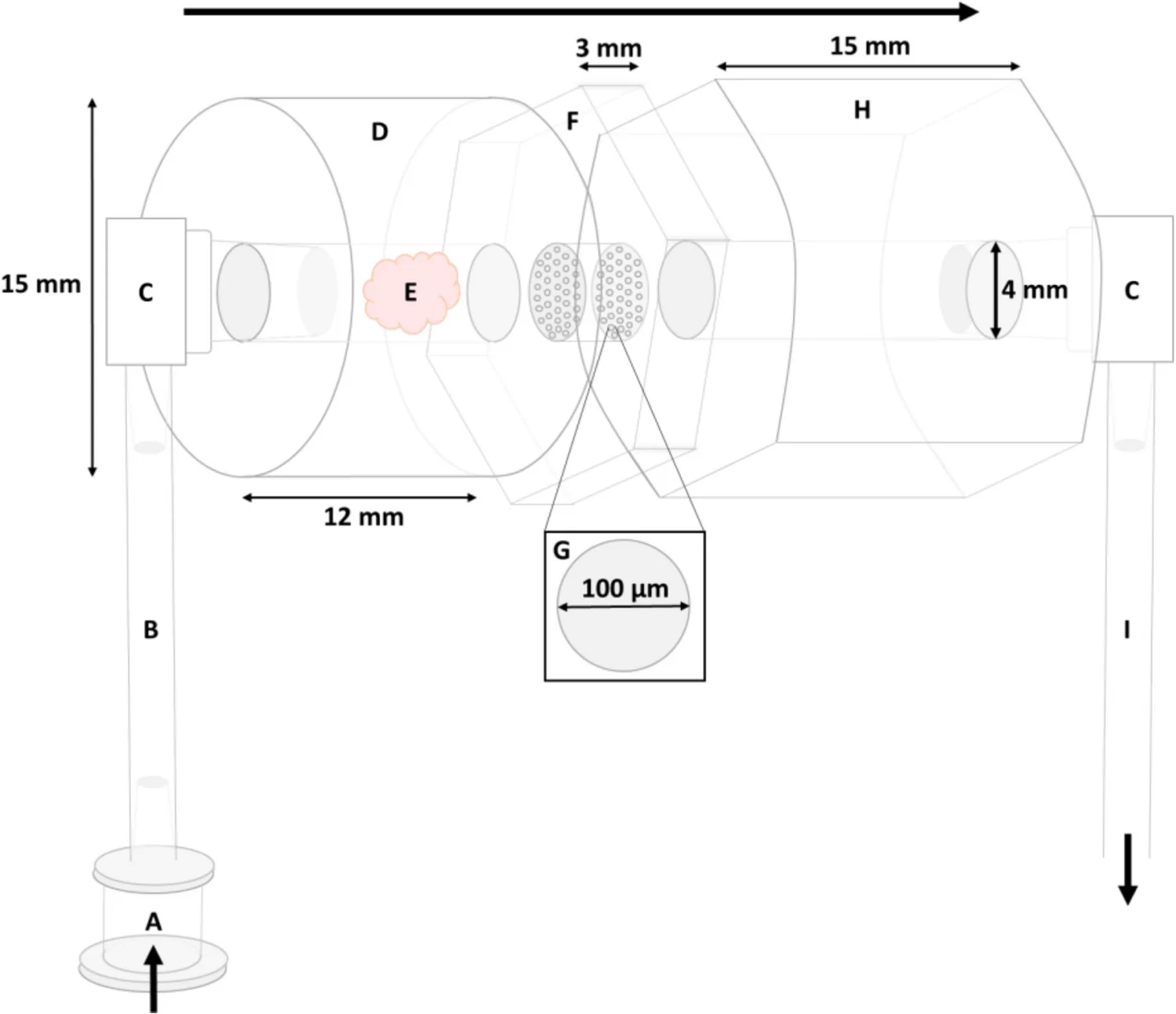
Diagram of the GBM-on-chip chamber. The chip is assembled from three polymethylmethacrylate (PMMA) components, fused together using chloroform. **A** Female Luer connector to attached to syringe-filter; **B** 1/32″ Tygon silicon inlet tube; **C** male Luer connectors, inserted into inlet and outlet chambers; **D** 12 mm × 15 mm inlet chamber, housing the tissue within 4 mm diameter internal chamber; **E** micro-dissected tissue (~ 3 mm diameter); **F** 3 mm × 15 mm central frit, permeability barrier, allowing media to flow through without losing the tissue; **G** 100 μm diameter pore within the permeability barrier; **H** 15 mm × 15 mm outlet chamber; **I** 1/32″ Tygon silicon outlet tube

Custom GBM-on-chip devices (20) were fabricated in-house from PMMA sheets that had been precision laser-cut and solvent-bonded with chloroform to form a single-chamber unit. The final assembly measured 30 mm in length consisting of three principal compartments: an inlet chamber measuring 12 × 15 mm, a central semipermeable barrier (“frit”) containing 37 pores of 100 µm in diameter and extending 3 × 15 mm, and an outlet chamber of 15 × 15 mm. Within the inlet chamber, a circular pocket of 4 mm diameter was designed to seat the tissue biopsy. Flow connections were established with 1/32″ Tygon 66 silicone tubing (Cole-Parmer, USA) fitted to male and female Luer connectors (Ibidi, Germany) (Fig. 1).

During assembly, small droplets of chloroform were applied to the mating surfaces to dissolve the PMMA, taking care to avoid blocking of the frit. The sections were pressed together for several seconds, allowing the chloroform to evaporate and fuse the pieces into a single unit. The assembled chips were then rinsed thoroughly and sterilised with 70% ethanol. Once connected to the tubing, the device provided a linear perfusion path from the syringe reservoir through the tissue chamber and permeability barrier, and out through the outlet tubing into sterile 15 ml polypropylene conical tubes (Corning, USA). The central porous barrier allowed dynamic delivery of treated medium and removal of waste, whilst preventing tissue loss through the outlet tube. Perfusion was maintained using a PHD ULTRA syringe pump (Harvard Apparatus, USA), which allowed precise control of flow rates and parallel operation of up to 10 chips. At a flow rate of 3 µl min^−1^, the mean linear velocity was calculated (Eq. 2.1) and the Reynolds number determined (Eq. 2.2), confirming laminar flow within the device.

***Equation 2.1*** ***: Calculation of the velocity (u):***

Relation between fluid velocity and the flow rate: Q = A.u

Q = flow rate (m^3^s^−1^) = 3 µl min^−1^ = 5.0 10^−11^ m^3^s^−1^

A = Area (m^2^) = π (d/2)^2^ = 1.25 × 10^−5^ m^2^

Q = A.u → u = Q / A = 4.0 × 10^−6^ ms^−1^

***Equation 2.2.: Calculation of Reynold number (Re):*** Re = ρ.u.d/µ [22] where:

ρ = Density of the medium (kg m^−3^) = 1007 kg m^−3^ [23]

u = Velocity of the liquid in the channel (ms^−1^) = 4.0 × 10^−6^ ms^−1^ (Eq. 2.1).

d = Diameter of the microchannel of the chip (m) = 4 mm = 4 × 10^−3^ m

µ = Dynamic viscosity (Nsm^−2^) = 9.4 × 10^−4^ Nsm^−2^ [24]

Re = 1.71 × 10^−2^

### Patient tissue handling

This project received Local Research Ethics Committee (LREC) approval (IRAS number 131630, LREC number: 13/YH/0238). Adult patients undergoing planned resection of suspected GBM at Hull University Teaching Hospitals NHS Trust were prospectively recruited and written, informed, consent was obtained prior to surgery. Intraoperatively, the neurosurgical consultant selected a non-necrotic portion of tumour, guided by intraoperative fluorescence with 5-aminolevulinic acid, and high-magnification microscopy; necrotic, cauterised, or vessel-rich regions were avoided.

Freshly excised tumour samples were immediately placed in 15 ml centrifuge tubes (Corning), containing complete Dulbecco’s Modified Eagle Medium (DMEM) (Sigma-Aldrich, #D5796) supplemented with 10% (v/v) foetal bovine serum (FBS) (Sigma-Aldrich #F7524), 1 mM sodium pyruvate (Gibco, #11,360,070), and 1X antimycotic/antibiotic cocktail (10,000 U penicillin, 1 mg streptomycin and 25 µg amphotericin B per ml) (Sigma-Aldrich, #A5955). The samples, typically weighing 0.25 g to 2.5 g, were transported in insulated containers to the University laboratory within 30–60 min.

All subsequent handling was carried out under sterile conditions in a Class II biosafety cabinet, at room temperature. Tumours were micro-dissected into uniform biopsies of approximately 20 mg (± 0.4 mg**,** about 3 mm in diameter, corresponding to ~ 14 mm^3^); each piece was weighed on a microbalance. For each patient, a ~ 20 mg tissue piece was processed immediately as a baseline reference by snap-freezing, a second was formalin-fixed and paraffin-embedded for future RNA and protein/histological analyses, respectively. Remaining pieces were used for perfusion experiments as described below.

### Perfusion culture protocol

Perfusion experiments were initiated prospectively as surgical specimens became available. A cohort of 13 patients was studied. Within each patient run, multiple devices were operated in parallel, with one biopsy per microfluidic device (up to ten devices per patient, depending on tissue availability). Replicates for each test condition were established and, when sufficient tissue was available, additional vehicle control (DMSO) and temozolomide (TMZ; 10 µM) conditions were set-up to enable within-patient comparisons.

Sterile syringes were filled with pre-warmed medium, with and without drug, connected to inline 0.2 µm filters, and attached to the inlet tubing. Prior to tissue loading, the system was primed with medium to remove air pockets and ensure continuous flow.

To load tissue, the outlet tubing was positioned vertically, the inlet tube was briefly disconnected, and a single fresh biopsy was placed in the central tissue pocket using sterile forceps. Residual air was displaced with medium, and the inlet connection was resealed. Devices were mounted on the syringe pump and maintained at 37 °C either inside a thermostatically controlled chamber or with the device chamber in an incubator while syringes remained on the pump. Flow was set at 3 µL min^−1^, generating a low-shear, laminar perfusion, regimen (calculated in Eqs. 2.1 and 2.2).

**Fig. 2:**
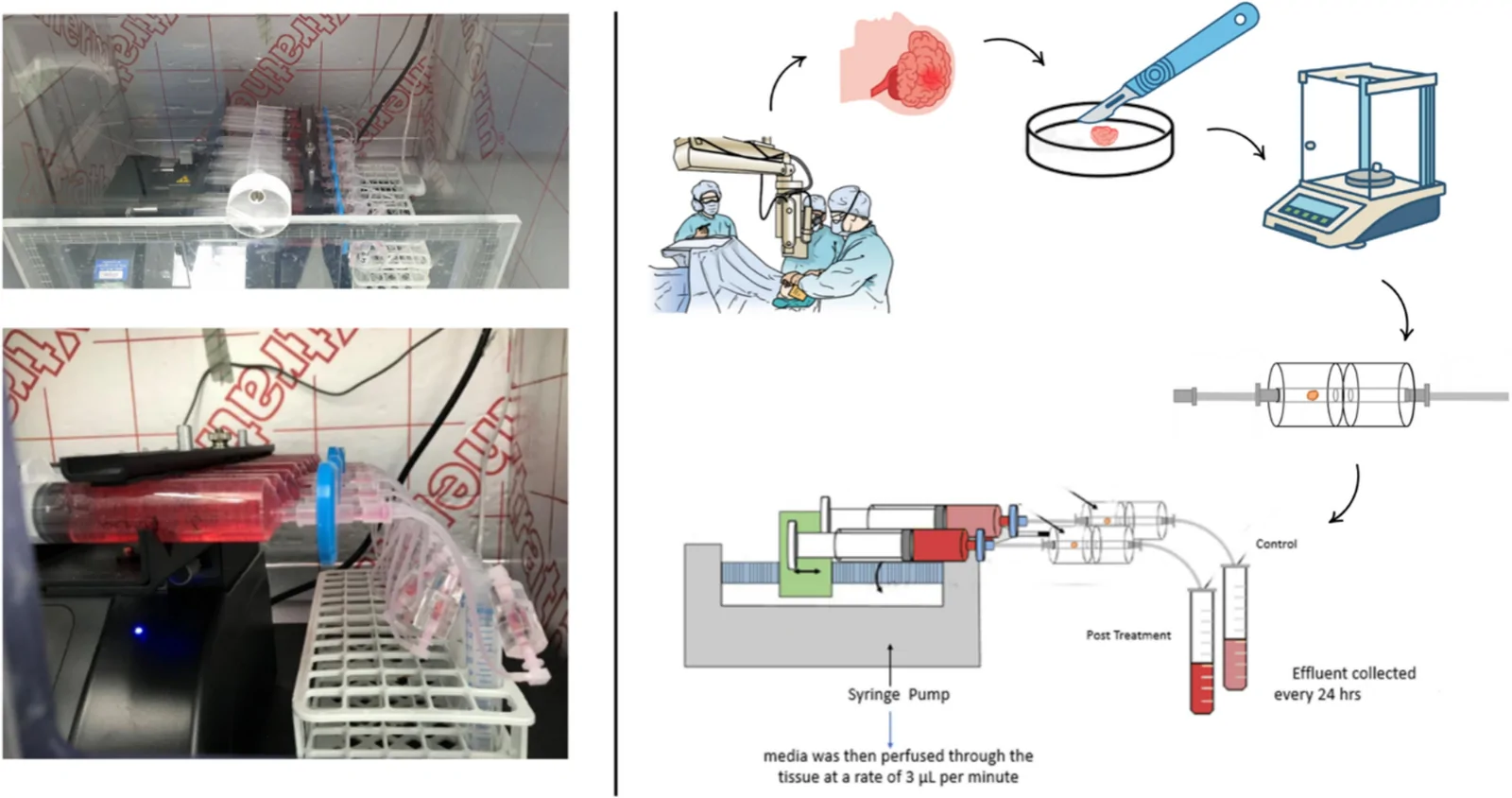
Illustration of the novel microfluidic setup: photograph (left) and design schematic (right). A single ~ 20 mg biopsy is seated in each perfusion device and perfused at 3 µL min^−1^ for up to 12 days with syringe changes on days 4 and 8. Effluent is collected every 24 h for downstream analyses, and tissue is preserved at baseline and post-perfusion

Perfusion experiments lasted 8 or 12 days. Syringes were replaced every 4 days using freshly prepared, pre-warmed medium. The system was checked regularly for leaks or blockages, and flow was visually verified. Effluent was collected at 24-h intervals for the duration of culture, and samples were stored at 4 °C for up to 12 days for LDH analysis or at − 80 °C once aliquoted for cytokine analyses (Fig. 2).

### Downstream outputs

The platform generated three complementary outputs. First, a matched pre-perfusion tissue sample provided baseline histology and molecular readouts (RNA and protein). Second, chip effluent captured the evolving tumour secretome and was analysed for metabolic viability via LDH release, broad cytokine screening using Proteome Profiler arrays, and targeted quantification of selected factors by ELISA. Cytokines for ELISA quantification were prioritised after review of the Proteome Profiler screen based on: (i) frequency of detection and abundance across effluent samples, (ii) evidence of treatment-associated modulation, and (iii) relevance to prior published work. These cytokines included: interleukin-6 (IL-6), interleukin-8 (IL-8), vascular endothelial growth factor (VEGF), angiopoietin-2 (ANGPT2), chitinase-3-like protein 1 (CHI3L1), serpin family E member 1 (Serpin-E1), and matrix metalloproteinase-9 (MMP-9). Finally, post-perfusion tissue was available for comparative histology and downstream molecular assays, e.g., RNA extraction for transcriptomic analysis and protein assays.

### Cytokine profiling and ELISA validation

Effluent samples were analysed using a Proteome Profiler XL Cytokine Array (R&D Systems) to survey 105 cytokines, with signals detected by chemiluminescence and quantified using QuickSpots software. Arrays were run on effluent collected at 48, 96 and 192 h (and 288 h in a subset of patients). To provide quantitative validation, selected cytokines were quantified by ELISA in duplicate, with concentrations interpolated from multi-point standard curves generated using a twofold serial dilution series using a 4-parameter logistic fit, prepared according to the manufacturer’s protocol.

### LDH activity assay

LDH activity in effluent was used as a marker of cellular damage/stress, as LDH is released upon loss of membrane integrity [25]. LDH activity was quantified in chip effluent collected at 24-h intervals using a colorimetric LDH assay kit (Sigma-Aldrich, #11,644,793,001) according to the manufacturer’s instructions. Effluent samples were assayed in duplicate, and absorbance was measured at 490 nm on a microplate reader with background subtraction using assay blanks’ LDH activity was finally normalised to biopsy mass (per mg tissue). Following completion of perfusion experiments, tissues were lysed using the Ultra Turrax T25 homogeniser, with 1% Triton-X100 and EDTA-free protease inhibitor, and the resulting supernatant analysed to assess total LDH activity.

### Histology: H&E

H&E staining was performed on matched baseline (T0, “time zero” tissue processed immediately after micro-dissection) and post-perfused GBM tissues. For 8-day perfusion samples, tissues were embedded in optimal cutting temperature (OCT) compound and frozen using methylbutane pre-chilled in liquid nitrogen. For 12-day samples, tissues were fixed overnight in 4% paraformaldehyde, dehydrated through graded alcohols and Histo-Clear (National Diagnostics), and embedded in paraffin wax prior to sectioning and H&E staining. Stained sections were imaged on an Olympus IX71 microscope using CellSens software (v1.18) and reviewed with a consultant neuropathologist.

### Immunohistochemistry and image analysis

Apoptosis markers (cleaved PARP and Annexin V) were assessed by immunohistochemistry on matched baseline (T0) and post-perfused tissues. Stained sections were imaged on an Olympus IX71 microscope using CellSens software (v1.18). Quantification was performed using an automated CellProfiler pipeline [26]. It was validated by technical replicates and manual review [20]. Sample sizes for each downstream analysis are summarised in Table 1.

**Table 1:** Sample size summary by analysis

Analysis / readout	Sample/material	Timepoints (hours, h or days, d)	Patients (n)	Biopsy-loaded chips (n)
LDH effluent time-course (8-day)	Effluent	Every 24 h to 192 h	12	12
LDH effluent time-course (12-day)	Effluent	Every 24 h to 288 h	4	4
H&E morphology / mitotic figures	Tissue sections	T0 vs post-perfusion (8 d / 12 d)	1	1
IHC apoptosis: cleaved PARP	Tissue sections	T0 versus 8 d; T0 vs 12 d	4	4
IHC apoptosis: Annexin V	Tissue sections	T0 versus 8 d	10	10
Proteome Profiler array (105 cytokines)	Effluent	48/96/192/ (288) h	13	26
Target ELISAs (selected cytokines)	Effluent	48/96/192/ (288) h	13	26

Sample sizes are reported as independent patients (biological replicates) and biopsy-loaded chips (one ~ 20 mg biopsy perfused per microfluidic device; one biopsy per chip). For longitudinal effluent assays, measurements represent repeated sampling from the same chips at the indicated timepoints. T0 = baseline (“time zero”) pre-perfusion tissue. Timepoints shown in parentheses were performed in a subset of patients only (288 h: n = 3, last three patients of the cohort)

### Statistical data processing and analysis

Proteome Profiler membranes were quantified in QuickSpots by averaging duplicate spots, subtracting background (negative controls), and normalising to positive controls to generate relative expression values; undetected signals were recorded as zero prior to transformation/normalisation. Statistical analyses were performed in R (v4.3.0). Cytokine datasets were transformed and normalised (MetaboAnalystR v4.0.0) prior to multivariate analysis. Group differences in multivariate cytokine profiles were assessed by PERMANOVA (vegan; 9999 permutations) using a distance matrix calculated from the transformed/normalised cytokine data, with permutations stratified by patient ID to account for repeated measures across timepoints; pairwise comparisons were performed where appropriate (pairwiseAdonis). Where indicated, univariate treatment effects were assessed using limma (moderated t-test) and/or paired tests as appropriate, with correction for multiple significance was defined as α = 0.05 (two-sided); adjusted p values are reported where applicable.

## Results

### Cellular stress remains low for up to 12 days of perfusion

**Fig. 3:**
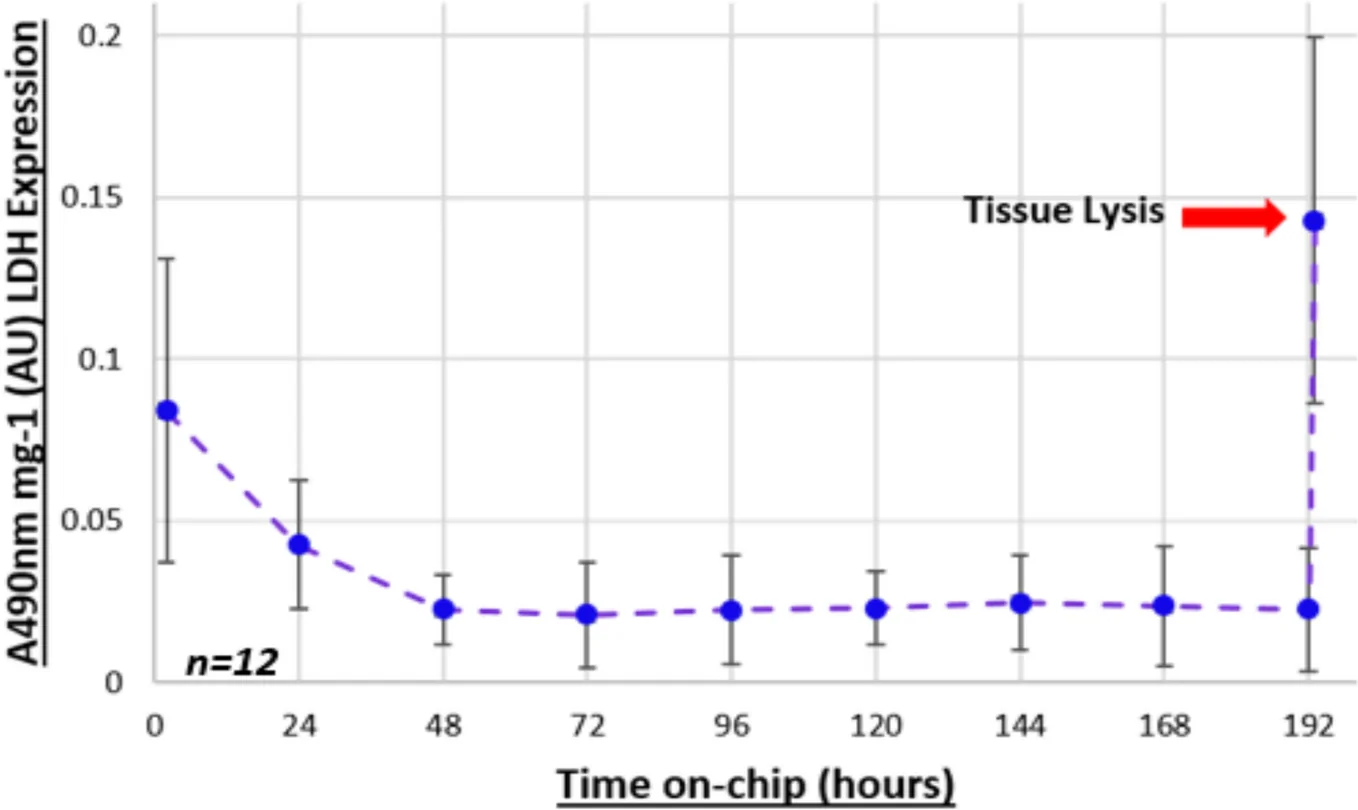
Lactate dehydrogenase (LDH) activity of GBM (n = 12) over 8 days. Effluent LDH activity (A490 mg^−^.^1^ tissue) measured over time; red arrow indicates end-point tissue lysis. Points show mean ± SD. Adapted from [20]

For the 8-day analysis, 12 different patient tissues were used. Initial LDH levels were high, most likely due to stress from tissue extraction and handling but decreased significantly over the first 48 h, stabilising at low levels (0.021–0.025 (absorbance units) AU mg⁻^1^) for the remaining days. There were no significant fluctuations in LDH activity over the incubation period, suggesting that the tissues remained viable in the perfusion device. After the 8-day period, the samples were lysed to quantify any retained LDH, which was a significantly larger amount compared to baseline expression (*t* = -6.64, df = 97, *p* = 1.80 × 10^−9^) (Fig. 3).

**Fig. 4:**
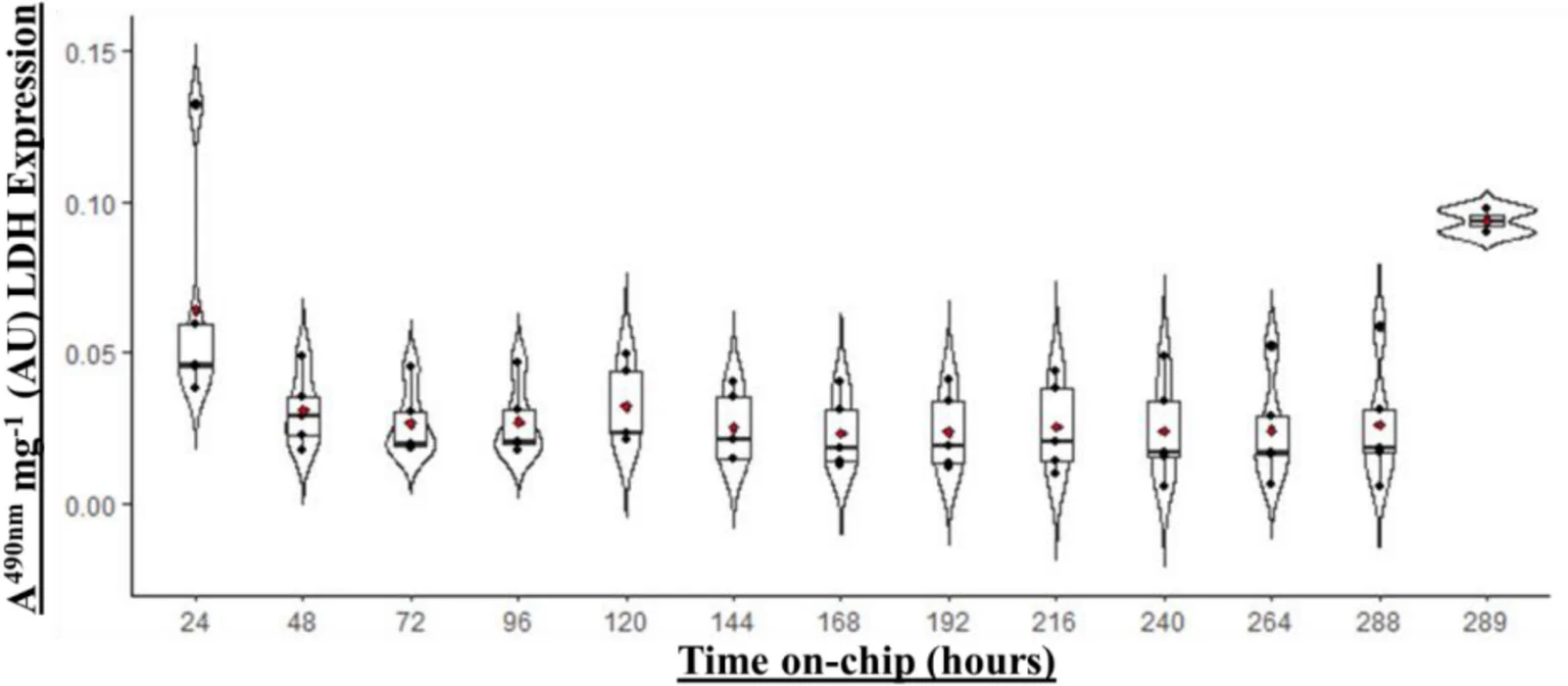
Lactate dehydrogenase activity in GBM tissue over 12 days. Effluent LDH activity (A490 mg^−1^ tissue) measured over time; end-point tissue lysis is shown at the final timepoint; n = 4. Points show mean ± SD

For the 12-day analysis, LDH was measured in four GBM samples. LDH activity showed an early decline within the first 48 h and then remained low thereafter (0.023–0.032 AU mg^−1^). Consistent with this, a one-way ANOVA detected no significant overall effect of time across the 12-day period (F = 1.38, df = 11, *p* = 0.22) (Fig. 4).

### Histological evidence of cell viability and mitosis

**Fig. 5:**
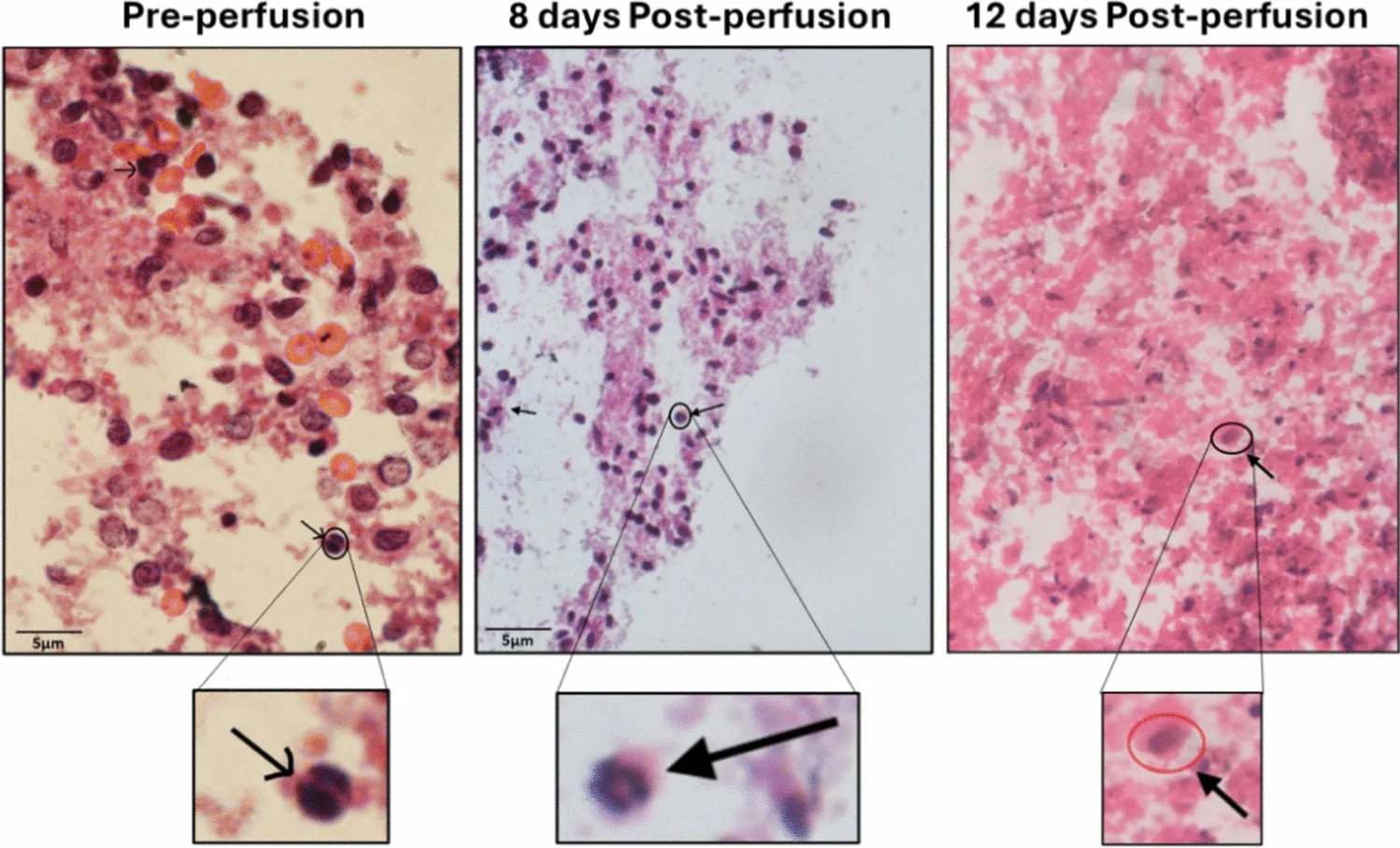
Representative H&E staining of GBM tissue at baseline (T0) and after 8 and 12 days of on-chip perfusion. Haematoxylin (purple) stains nuclear aspects of cells and eosin stain highlights cytosolic cellular fractions. Arrows on both images highlight mitotic figures, with inset showing magnified images. Scale bars as indicated; images acquired at × 40 magnification

H&E staining of matched baseline (T0) and post-perfused GBM tissues at 8 and 12 days was used to assess tissue architecture (Fig. 5). Microscopic examination identified mitotic figures in both pre- and post-perfused tissues, indicating that tumour cells remained capable of division after on-chip perfusion. These observations are consistent with preserved tissue viability and proliferative capacity over the experimental period.

The presence of unbalanced mitosis and chromatin distribution in some cells further confirmed that these were tumour cells actively proliferating, rather than non-tumour contaminant cells. This histological evidence supports the conclusion that GBM tissues maintained their viability and proliferative capacity within the perfusion device over the experimental period [20].

### Assessment of apoptosis post-perfusion (cleaved PARP and Annexin V)

**Fig. 6:**
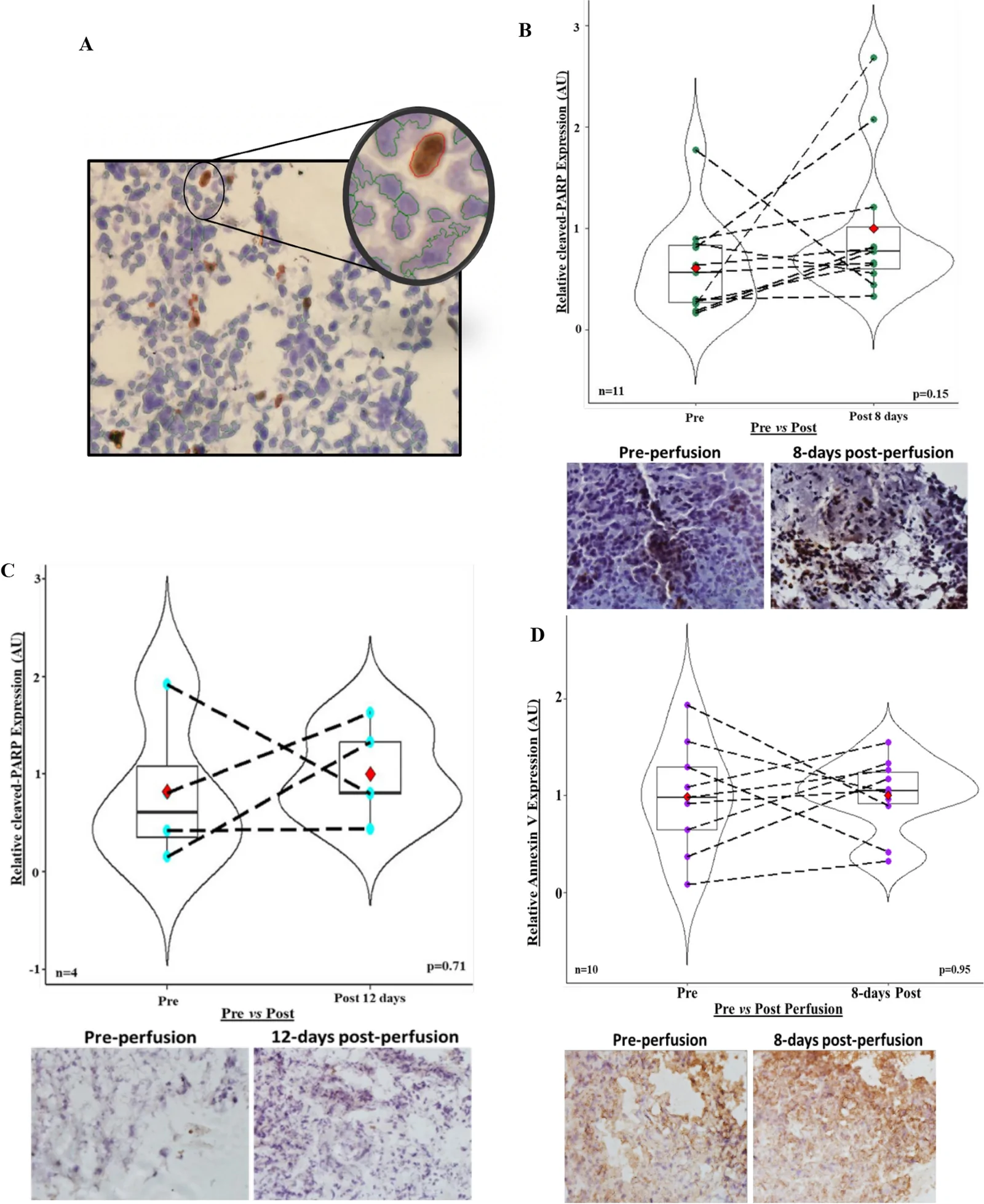
Apoptosis markers in GBM tissue before and after on-chip perfusion. Immunohistochemistry for cleaved PARP and Annexin V in matched baseline (T0) and post-perfused tissues. Values are expressed relative to the post-perfusion sample within each patient pair (post-perfusion = 1). Representative images show haematoxylin (nuclei, blue) and DAB (positive staining, brown). Dashed lines indicate paired patient samples. **A** Example of automated detection of cleaved PARP-positive cells. **B** Cleaved PARP: T0 versus 8 days (n = 11; paired Mann–Whitney U, *p* = 0.151). **C** Cleaved PARP: T0 versus 12 days (n = 4; paired t-test, p = 0.71). **D** Annexin V: T0 versus 8 days (n = 10; paired t-test, *p* = 0.95)

To ascertain whether perfusion induced apoptosis in GBM tissues, cleaved PARP and Annexin V staining were compared between baseline (T0) and post-perfused tissues at 8 and 12 days (Fig. 6). Cleaved PARP showed that no statistically significant difference was observed between the two tissues at 8 days or 12 days. Similarly, Annexin V staining showed no significant changes in apoptosis between pre- and post-perfused tissues at 8 days.

Together, these findings indicate that the microfluidic perfusion process did not measurably increase apoptosis over the experimental period, further supporting tissue viability on-chip.

### Cytokine secretion confirms sustained viability and functional activity of GBM biopsies on-chip

#### A. Time-dependent modulation of cytokine secretion

**Fig. 7:**
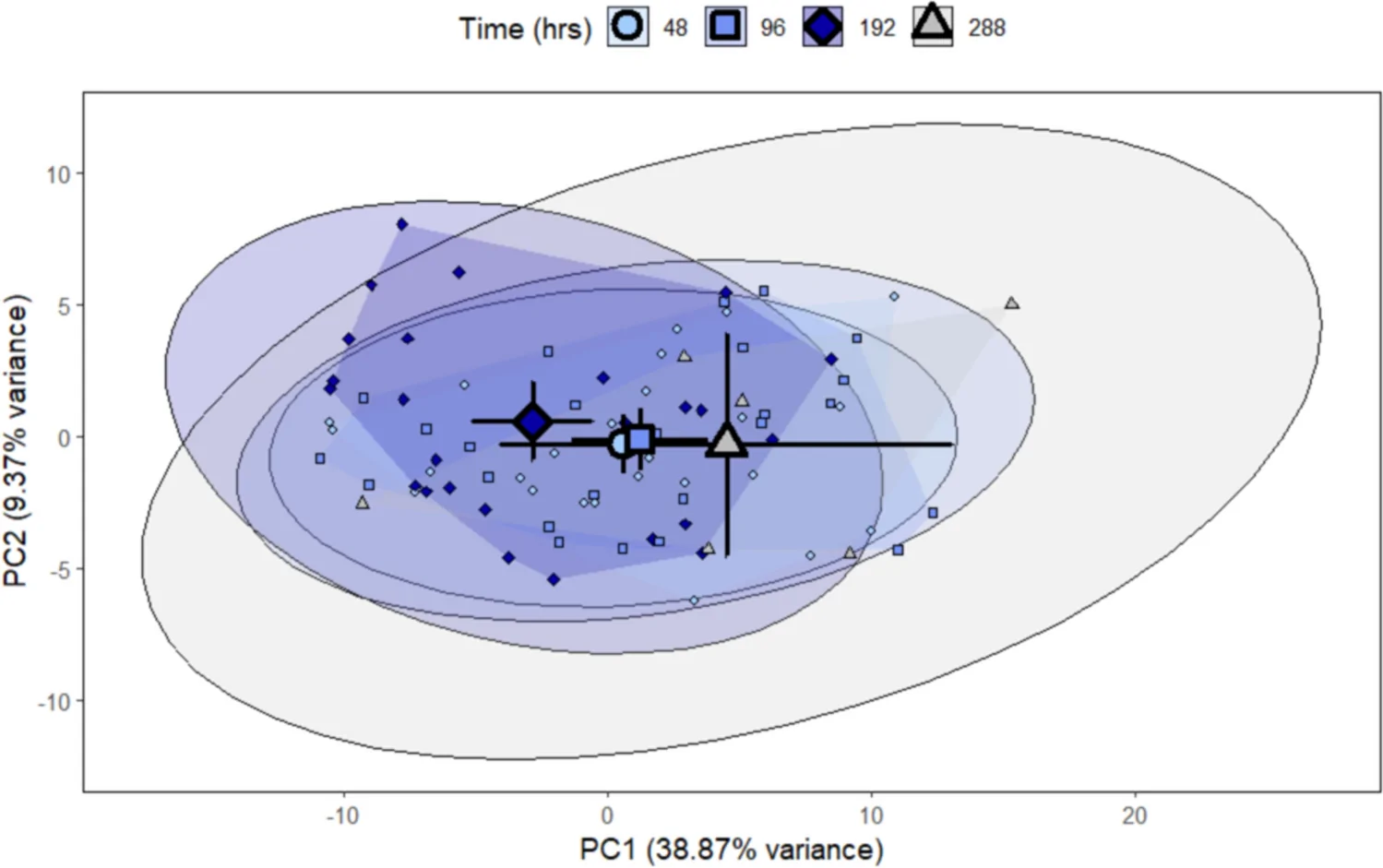
Principal component analysis of cytokine profiles over time (m = 105). Centroids (± 95% confidence intervals) and individual samples are shown for each timepoint; ellipses indicate 95% confidence intervals. Polygons show the maximal dispersion between outermost points per treatment

A further indicator of GBM tissue viability on the microfluidic platform was the sustained secretion of a broad panel of cytokines into the effluent across multiple time points up to 12 days (192 h). The divergence observed at 192 h reflected a broad multivariate shift across the 105-cytokine Proteome Profiler dataset rather than a change confined to the seven cytokines selected for ELISA validation. Pairwise PERMANOVA showed that the 192 h timepoint differed significantly from both 48 and 96 h, whilst 48 and 96 h were not significantly different from each other. Overall, most cytokines showed lower abundance at later time points. Individual treatment-associated cytokines were explored using partial least squares discriminant analysis (PLS-DA) and limma modelling with covariate adjustment. Although several cytokines showed nominal differences, only Angiopoietin-2, Apolipoprotein A-I and BDNF remained significant after false discovery rate (FDR) correction. An exploratory analysis including a 288 h timepoint (subset n = 3 patients) indicated further separation from earlier timepoints, consistent with the progressive modulation observed in the PCA (Fig. 7; Table 2).

**Table 2:** Pairwise PERMANOVA results for the effect of time on cytokine profiles

Timepoint comparison	F (PERMANOVA)	R^2^ (time)	*p*
48 h versus 96 h	0.63	0.01	0.8465
48 h versus 192 h	2.09	0.04	**0.0381**
96 h versus 192 h	3.09	0.05	**0.0117**
48 h versus 288 h (subset n = 3)	2.26	0.07	**0.0298**
96 h versus 288 h (subset n = 3)	2.21	0.06	**0.0423**
192 h versus 288 h (subset n = 3)	3.62	0.10	**0.0049**

(Proteome Profiler, m = 105); significant *P* values highlighted in bold

These findings indicate that, while initial cytokine release patterns remained relatively consistent, GBM tissues underwent measurable secretory shifts after 192 h and even more prominently at 288 h, reflecting ongoing modulation of cytokine secretion of tissue within the microfluidic environment.

#### B. Treatment responsiveness validated by ELISA quantification

To provide quantitative follow-up of the 105-cytokine screen, we selected seven cytokines for ELISA quantification: VEGF, MMP9, CHI3L1, ANGPT2, IL-6, IL-8 and Serpin-E1. Selection was based after a comprehensive review, prioritising cytokines that were abundant and/or frequently detected across effluent samples, or that showed evidence of treatment-associated modulation. IL-6 was additionally included a priori based on previous work in our laboratory that has shown it to be highly expressed in GBM. ELISA analysis demonstrated that treatment with TMZ significantly influenced the overall profile of this seven-cytokine panel (PERMANOVA, *p* = 0.039; n = 7). At the individual cytokine level, VEGF decreased significantly with treatment (P-adj = 0.0011), whereas CHI3L1 (P-adj = 0.0239) and ANGPT2 (P-adj = 0.0239) increased; IL-6, IL-8, MMP9 and Serpin-E1 did not show significant treatment-associated changes with treatment after FDR correction. Together, these findings indicate that the microfluidic platform preserved a measurable and treatment-responsive secretory phenotype in ex vivo GBM biopsies (Table 3).

**Table 3:** ELISA cytokine expression by treatment

Cytokine	Log_2_ FC	Direction upon TMZ treatment	AveExpr	T	P	P-adj	B
**VEGF**	** − 1.24**	**↘ in treatment**	**0.31**	** − 3.97**	**0.0002**	**0.0011**	**0.67**
**Angiopoietin.2**	**0.64**	**↗ in treatment**	**0.15**	**2.76**	**0.0072**	**0.0239**	** − 2.79**
**Chitinase3.Like.1**	**0.92**	**↗ in treatment**	**0.25**	**2.63**	**0.0102**	**0.0239**	** − 3.1**
IL-6	** − **0.18	Nonsignificant	0.29	** − **0.51	0.6088	0.8401	** − **6.23
MMP9	** − **0.09	Nonsignificant	0.18	** − **0.38	0.7037	0.8401	** − **6.29
IL-8	0.16	Nonsignificant	0.39	0.36	0.72	0.8401	** − **6.3
Serpin.E1	** − **0.1	Nonsignificant	0.44	** − **0.18	0.8596	0.8596	** − **6.35

(n = 7; 48/96/192 h). logFC = log2 fold-change (treated vs control). P-adj = adjusted p value. Significant cytokines after FDR correction are shown in bold

#### C. Cross-validation between Proteome Profiler and ELISA measurements

**Fig. 8:**
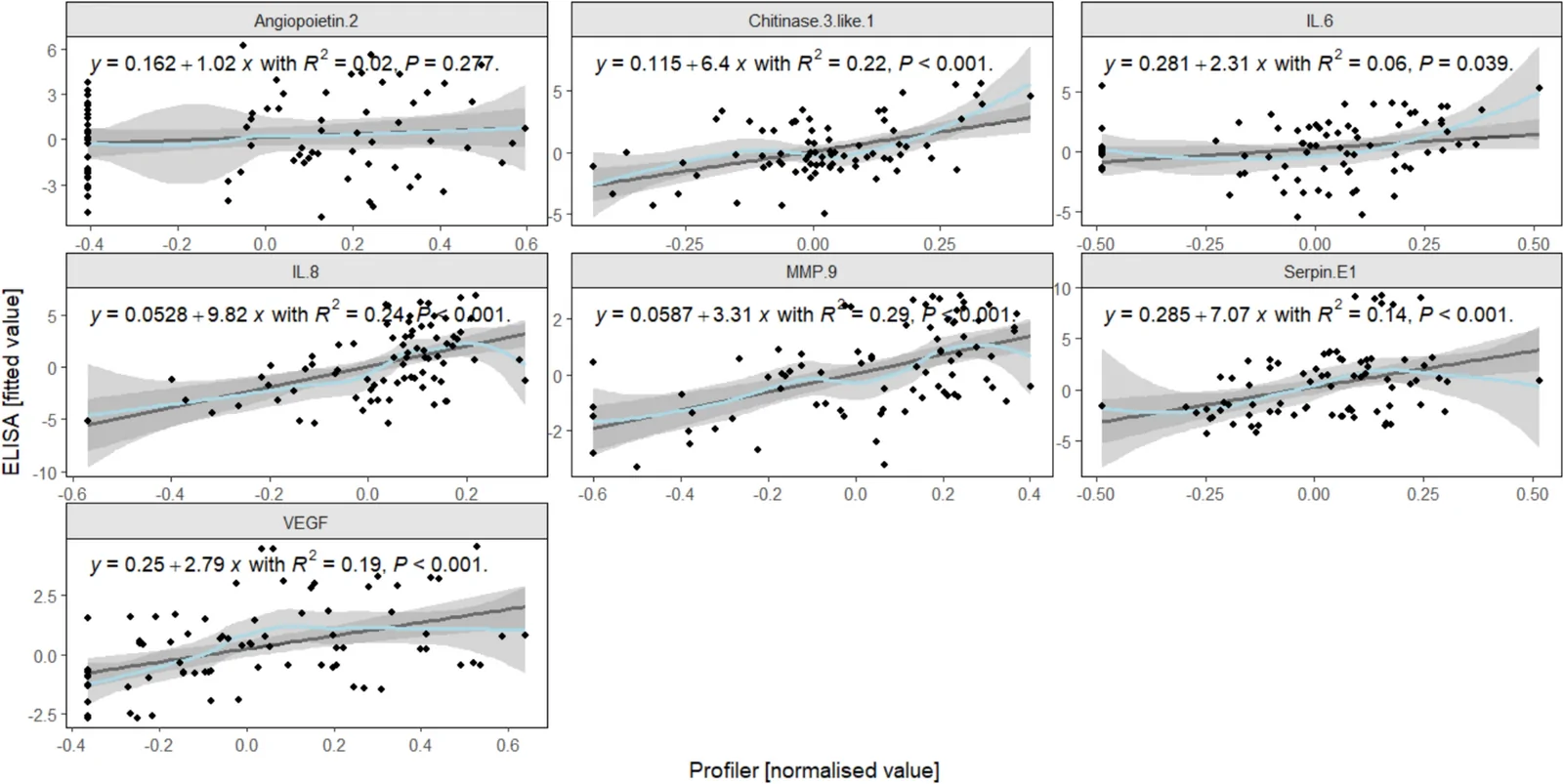
Correlation between Proteome Profiler and ELISA measurements for the targeted cytokine biomarker panel. Scatter plots show transformed/normalised Proteome Profiler values versus ELISA concentrations for matched effluent samples (48, 96 and 192 h). Grey lines indicate least squares linear fits (± 95% confidence intervals). Pearson r, R^2^ and two-sided *p* values are shown in each panel, together with the fitted slope equation

To assess concordance between the two analytical methods, cytokine concentrations measured by ELISA were compared with their corresponding transformed/normalised Proteome Profiler values for the selected cytokine biomarker panel. Six of seven cytokines exhibited significant positive correlations between the two independent methods (R^2^ = 0.06–0.29, *p* < 0.05), supporting data accuracy. The highest correlations were observed for MMP9 (R^2^ = 0.29, *p* < 0.001), CHI3L1 (R^2^ = 0.22, *p* < 0.001), and VEGF (R^2^ = 0.19, *p* < 0.001), whereas IL-6, IL-8, and Serpin-E1 showed weaker but significant associations. Angiopoietin-2 did not show a significant correlation (R^2^ = 0.02, *p* = 0.277). In the Proteome Profiler dataset, values below the detection threshold are recorded as zero and appear at the lower bound of the transformed scale (e.g., ~  − 0.4), producing a floor effect that can reduce correlation; therefore, this comparison is interpreted cautiously (Fig. 8).

## Discussion

We used a PMMA-based GBM-on-chip perfusion platform for ex vivo maintenance of intact, patient-derived biopsies. Using complementary readouts (LDH activity, histology/mitosis, apoptosis markers, and longitudinal secretome profiling), tissues remained viable and functionally secretory for up to 12 days, with treatment-associated modulation detectable in a targeted cytokine biomarker panel.

PMMA-based devices have emerged as a robust alternative to polydimethylsiloxane (PDMS) for tumour-on-a-chip applications. Kang’s group developed one of the first tissue-on-chip devices using PMMA, which is impermeable to small lipophilic molecules. In their comparative experiments with human lung adenocarcinoma cells, PMMA-based chips produced more reliable and reproducible cytotoxicity results after vincristine exposure than PDMS-based chips [27]. Beyond its performance advantages, PMMA is recyclable and reusable following simple sterilisation, making it an environmentally sustainable option [28]. It also provides excellent optical clarity, mechanical stability, and scalability through automated laser micromachining [29, 30]. A previous study from our laboratory employed the same PMMA-based microfluidic perfusion device to maintain patient-derived GBM biopsies in a viable state for up to eight days [20] focussing on drug response to protein arginine methyltransferase inhibitors. The current study extends the culture period to twelve days and shifts the emphasis from drug-induced molecular pathways to long-term tissue viability, and dynamic cytokine secretion.

Most microfluidic applications for brain tumours have focused on glioma, as it is the most common and deadly primary brain tumour. An early study developed a microfluidic device consisting of four parallel chambers demonstrated the feasibility of using simple microfluidic platforms for drug testing, finding that [31] increasing colchicine concentration or treatment time induced marked changes in cell morphology and increased cell death in C6 rat glioma cells. A 3D “brain cancer chip” fabricated from the hydrogel Polyethylene Glycol Diacrylate (PEGDA), with top and bottom glass covers, for high-throughput drug screening (Pitavastatin and Irinotecan) using U87 GBM spheroids has also been described [32]. In this system, spheroids were grown over 7 days (300–400 µm diameter) and subsequently exposed to drug combinations for a further 7 days. However, this approach relies on cell-line spheroids rather than intact patient tissue.

Xiao et al. [18] developed a vascularised microfluidic model to investigate the behaviour of brain tumour stem-like cells (BTSCs) within a perivascular niche-like environment. Dissociated BTSCs from ten primary IDH-wildtype GBM patients were co-cultured with GFP-labelled human umbilical vein endothelial cells in a commercial AIM Biotech PDMS device; this device consists of a central hydrogel channel flanked by two lateral media channels. After 48 h of culture, live confocal microscopy showed that BTSCs displayed heterogeneous behaviours: some cells remained quiescent, whereas others showed active migration mimicking in *vivo* infiltration. Together, these studies demonstrate elegant short-term microfluidic assays of tumour behaviour but generally operate over hours to a few days and use dissociated cells.

The first microfluidic device to utilise intact patient-derived GBM tissue was developed by the Hull group [21]. Using 128 tissue biopsies from 33 patients, they demonstrated that GBM tissue (10–15 mg / device) can be viably maintained in a PDMS–glass microfluidic chip for approximately 72 h. More recently, a microfluidic platform called MAqCI (Microfluidic Assay for Quantification of Cell Invasion) was designed to mimic GBM invasion through brain tissue [19]. Primary GBM cells freshly isolated from treatment-naïve surgical resections were briefly cultured and then seeded into laminin-coated channels to simulate the brain ECM. Migration was monitored by 24-h live-cell imaging, and proliferation was assessed using Ki-67 staining. The assay classified 28 patients into short- *vs.* long-term progression-free survival groups with 86% accuracy and predicted time to recurrence. The combined MAqCI score, based on migratory and proliferative behaviour, outperformed traditional prognostic markers, including IDH1 mutation status providing a rapid, low-cost and clinically informative assay for GBM prognosis. Together, these studies illustrate the diversity of microfluidic approaches for modelling glioblastoma, from 2D drug-response chips and 3D spheroid platforms to vascularised BTSC models, intact tissue perfusion devices and invasion assays.

An additional advantage of this biopsy model over many published microfluidic GBM systems is the use of intact human surgical specimens rather than cell lines, engineered constructs or pre-formed spheroids. It reduces the need for in vitro expansion and enzymatic dissociation, preserves intra-tumoural heterogeneity, and allows integration of clinically relevant covariates into the cytokine analyses. It also allows for multiple dynamic downstream readouts, such as Proteome Profiler and ELISA, as well as histology, rather than single end-point assays, which distinguishes this microfluidics model from other platforms discussed (see Table 1). The seven cytokines selected for ELISA validation were chosen because they were abundant and/or frequently detected in the Proteome Profiler screen, showed evidence of treatment-associated modulation, or were of prior interest based on previous work. Collectively, this panel includes a range of processes critical to GBM development, including angiogenesis and vascular remodelling (VEGF, Angiopoietin-2; [33–35]), extracellular matrix remodelling and invasion (MMP9; [36]), inflammatory signalling (IL-6, IL-8; [37]), a mesenchymal associated phenotype (CHI3L1; [38]), and tissue remodelling or stress-associated signalling (Serpin-E1; [39]). However, the observed treatment-associated changes should be interpreted as exploratory functional readouts of the platform rather than definitive mechanistic evidence of TMZ response; this would require a large, appropriately powered, study. As such, the platform sits between highly reductionist in vitro models and more complex, costly in vivo approaches, and is well placed for translational and personalised applications. The main limitation of the current study is the relatively small sample size (n = 13), which restricts generalisability and means the findings should be considered exploratory. Not all biopsies were maintained for the full 12-day duration; most experiments were intentionally completed at 8 days as a planned end point due to resource constraints, rather than evidence of tissue decline or device failure. A subset was extended to 12 days to assess longer-term stability, and longer culture periods will be explored in future work.

By enabling dynamic profiling of cytokine release from live patient-derived GBM tissue under defined treatment conditions, this microfluidic platform has potential clinical relevance as a patient-specific testing system. In principle, it could be used to assess individual tumours’ functional responses to standard-of-care therapies and novel combinations, informing personalised treatment decisions and stratification in early-phase trials. Data from larger cohorts would allow correlation of all the standard clinical markers with the longitudinal secretome data, which would support exploratory biomarker discovery. Matrix-based invasion compartments, paired with serial imaging and volumetric tissue assessment, as well as utilisation of hypoxia-controlled incubators and assessment of hypoxic markers, e.g., HIF1α, could also be incorporated into the platform hopefully reducing reliance on animal models whilst generating more clinically meaningful data.

## Conclusion

Microfluidic chips provide a cost-effective and physiologically relevant environment for GBM research by replicating in vivo-like perfusion and reducing sample and reagent use. Our work demonstrates the feasibility of maintaining fresh patient-derived GBM tissue on a PMMA-based microfluidic platform for up to 12 days, with preserved tissue viability and measurable, treatment-responsive cytokine secretion. Compared with spheroid or organoid approaches, the system offers a closer approximation of the native tumour microenvironment while remaining compatible with multiplexed biochemical and immunohistochemical readouts. With further optimisation, scaling and multicentre validation, this platform has the potential to become a useful tool for preclinical therapeutic screening, biomarker discovery and, ultimately, personalised medicine in neuro-oncology.

## Data Availability

The datasets generated and/or analysed during the current study are available from the corresponding author on reasonable request. Patient-derived data are not publicly available because of ethical and privacy considerations.
